# Elevation of GABA levels in the globus pallidus disinhibits the thalamic reticular nucleus and desynchronized cortical beta oscillations

**DOI:** 10.1186/s12576-022-00843-3

**Published:** 2022-07-27

**Authors:** Nelson Villalobos, Salvador Almazán-Alvarado, Victor Manuel Magdaleno-Madrigal

**Affiliations:** 1 Academia de Fisiología, Escuela Superior de Medicina, Instituto Politécnico Nacional, Plan de San Luis y Díaz Mirón, Colonia Casco de Santo Tomás, 11340 Ciudad de México, Mexico; 2 Sección de Estudios de Posgrado e Investigación de la Escuela Superior de Medicina, Instituto Politécnico Nacional, Plan de San Luis y Díaz Mirón, Colonia Casco de Santo Tomás, 11340, Ciudad de México, Mexico; 3 Laboratorio de Neurofisiología del Control y la Regulación, Dirección de Investigaciones en Neurociencias, Instituto Nacional de Psiquiatría Ramón de la Fuente Muñiz, Ciudad de México, Mexico; 4 Carrera de Psicología, Facultad de Estudios Superiores Zaragoza-UNAM, Ciudad de México, Mexico

**Keywords:** Motor control, Globus pallidus, Beta band, Tonic inhibition, Reticular thalamic nucleus, Synchronization

## Abstract

The external globus pallidus (GP) is a GABAergic node involved in motor control regulation and coordinates firing and synchronization in the basal ganglia–thalamic–cortical network through inputs and electrical activity. In Parkinson's disease, high GABA levels alter electrical activity in the GP and contribute to motor symptoms. Under normal conditions, GABA levels are regulated by GABA transporters (GATs). GAT type 1 (GAT-1) is highly expressed in the GP, and pharmacological blockade of GAT-1 increases the duration of currents mediated by GABA A receptors and induces tonic inhibition. The functional contribution of the pathway between the GP and the reticular thalamic nucleus (RTn) is unknown. This pathway is important since the RTn controls the flow of information between the thalamus and cortex, suggesting that it contributes to cortical dynamics. In this work, we investigated the effect of increased GABA levels on electrical activity in the RTn by obtaining single-unit extracellular recordings from anesthetized rats and on the motor cortex (MCx) by corticography. Our results show that high GABA levels increase the spontaneous activity rate of RTn neurons and desynchronize oscillations in the beta frequency band in the MCx. Our findings provide evidence that the GP exerts tonic control over RTn activity through the GP–reticular pathway and functionally contributes to cortical oscillation dynamics.

## Introduction

The reticular thalamic nucleus (RTn) articulates oscillations in large neuronal assemblies [15, 31]. GABAergic neurons in the RTn receive collaterals from thalamocortical (TC) and corticothalamic (CT) fibers and these connections modulate information transfer between thalamic nuclei and the cerebral cortex [27, 69]. The RTn also receives inputs from noradrenergic, serotonergic, and cholinergic clusters in the brainstem [2, 4, 14, 29, 62]. These afferents control electrophysiological properties and regulate all TC activity. In this way, the RTn in combination with its afferents induces oscillations associated with functions such as attention, sleep, and consciousness [10, 15, 80]. Alterations in these oscillations are related to the initiation of several brain diseases [10, 20, 64, 78].

The topographic organization of projections results in the formation of functional sectors in the RTn: one limbic sector, five sensory sectors, and one motor sector [12, 39]. In rodents, the motor sector is in the rostral region of the RTn [26, 67, 69] and receives GABAergic projections from the globus pallidus (GP; external GP in primates) [3, 17, 24, 35, 41]. However, the functional implications of the GP–RTn network, especially in motor control, have not yet been clarified. The ability of the GP to modulate the electrical activity of RTn neurons in vivo [65, 83] and the involvement of the RTn in locomotor activity [53] were recently reported.

In the context of this experimental evidence, the GP–RTn pathway has attracted our attention for two reasons. First, classical models of basal ganglia (BG) circuits assert that output nuclei of the circuits (the internal GP and substantia nigra pars reticulata) are the principal connections to motor nuclei of the thalamus (Th) and therefore regulate information flow toward the cortex [1, 19, 25]. This model does not consider the involvement of the GP–RTn pathway in the general function of the BG-thalamic circuit. Second, the GP was recently revealed to be a key nucleus in BG functions [43, 52], and electrical activity in the GP is altered in movement disorders such as Parkinson's disease [7, 33, 60]. Pathophysiologically, this abnormal GP activity has been related to alterations in GABA levels [19].

According to this association, the normal function of the GP within the BG depends on the appropriate regulation of environmental GABA levels [22]. The activity of GABA transporters (GATs) is essential for achieving such regulation [18, 40, 70]. Once released by presynaptic terminals, GATs rapidly remove GABA from the extracellular space; thus, in addition to modulating inhibitory synaptic transmission [6, 70], GATs regulate the leakage of GABA into neighboring synapses [6, 61] and, by maintaining GABA homeostasis, prevent excessive tonic activation of synaptic and extrasynaptic GABA receptors [6, 75].

In rodents, GAT type 1 (GAT-1) and 3 (GAT-3) are expressed at high levels in the GP [23, 37]. Functionally, intrapallidal administration of a GAT-1 antagonist significantly increases environmental GABA levels and reduces the rate of pallidal firing [22]. In addition, pharmacological blockade of GAT-1 or GAT-3 prolongs the duration of synaptic currents mediated by GABA A receptors and induces persistent tonic inhibition in the GP [37]. In this contextual framework, we hypothesize that pharmacological elevation of pallidal GABA levels affects spontaneous activity in the RTn by activating the GP–reticular pathway and that this alteration affects cortical oscillations. Because electrical activity in the GP is key during motor control and since we record from to rostral portion of the RTn, our data suggest that the RTn controls TC activity via tonic effects on the GP–RTn pathway in the beta frequency band.

## Experimental procedure

### Subjects

Experiments were performed on male Wistar rats 8–12 weeks old. The rats were maintained in individual cages in a room with an ambient temperature of 20–24 °C on a 12/12 h dark cycle and given free access to water and food. The rats were maintained and handled according to the ESM-IPN guidelines (based on the standards on the care and use of animals for experimental procedures published by the US National Institute of Health) and the local Animal Ethics Committee of Instituto Nacional de Psiquiatría Ramón de la Fuente Muñiz. All of the experimental procedures followed the Norma Official Mexicana for the care and use of laboratory animals (NOM-062-ZOO-1999). Efforts were made to minimize the number of animals used and their suffering.

### Surgery

During surgery, the rats were anesthetized by intraperitoneal injection of 1.25 mg/kg urethane (Sigma-Aldrich); subsequently, they were placed in a stereotaxic frame (David Kopf, Tujunga CA, USA), and their body temperature was maintained between 37 and 38 °C with a heating pad and rectal thermometer system (Frederick Haer, Bowdoin ME, USA). After an incision was made in the skull, two burr holes were made, the recording electrode was implanted in the RTn and the cannula guide system was implanted in the GP. The recording electrode was implanted at the following coordinates: 1.4 mm posterior to bregma, 1.2–2.1 mm lateral to bregma, and 5.3–7 mm deep relative to the dura. The cannula was implanted in the core of the GP at an angle of 60° relative to the horizontal in the lateral plane at the following coordinates: 0.8 mm AP, 5.8 mm lateral to bregma, and 5.8 mm deep concerning the dura mater. Electrocorticograms (EcoGs) were recorded from the motor cortex (MCx) by implanting steel screws 3.70 mm anterior to bregma and 1.9 mm lateral to the midline and a grounding electrode above the parietal bone. All stereotaxic coordinates were determined using a rat brain atlas [63].

### Electrophysiology

Extracellular recordings were made from the RTn using glass micropipettes filled with 2 M NaCl with a resistance of 5–10 MΩ; during recording, tracks were oriented vertically according to predetermined coordinates. The signals were amplified 10,000x, bandpass filtered between 0.3 and 3 kHz (DAM-80 WPI, Sarasota FL, USA), and saved to a PC for thorough offline analysis. The change in the spontaneous firing rate of RTn neurons after drug infusion into the GP was considered significant if it exceeded two standard deviations of the firing rate in the control period within 30 s after drug infusion. Once these parameters were established, the coefficient of variation (CV) was calculated as the ratio of the standard deviation of the interspike interval (ISI) to the mean of the ISI. ECoG signals were amplified, bandpass filtered (1–100 Hz), and digitized (300 samples/s). Spectral analysis of recording data from 5-s periods was performed by fast Fourier transformation (Hanning window function; data point block size of 1024; resolution of 0.9766 Hz).

Due to the involvement of the GP in beta oscillations of 10–30 Hz, the analysis was focused on this frequency range. To avoid the presence of artifacts, prior to analysis, the recording data were digitally filtered (bandpass: 5–50 Hz) in each time window. Subsequently, power spectra were calculated in the frequency band of interest, and the results for each neuron were averaged. The same approach was applied under basal conditions and after pallidal infusion. To assess the relationship between MCx and RTn activity in the same frequency range, coherence analysis (the same window, block, and resolution parameters as for FFT) was used. The data were analyzed similarly to the data used for power spectra estimation. Spectrograms were generated with a custom-written program in MATLAB (2020b MathWorks, Natick, MA, USA). Spike 2 analysis software (Cambridge Electronic Design, Cambridge, UK) was used for offline analysis.

### Drugs

Tiagabine (tiagabine hydrochloride, Sigma-Aldrich) and nipecotic acid (Sigma-Aldrich) were dissolved in 0.9% w/v NaCl saline immediately before use. The drug solution was injected unilaterally into the GP during RTn recording, and only neurons that exhibited stable baseline firing for five minutes were selected. The total injection volume for each infusion was 100 nl. The cannula guide system (30 gauge) was connected to a microsyringe (Hamilton, 10 µl) through a polyethylene tube and connected to a precision micrometer head, and infusion was performed at a rate of 50 nl/15 s. A single application was made in each rat. However, only in some cases was more than one application made. In these cases, the injections were separated by at least 1 mm and with an application interval of 10 min.

### Histology

At the end of the trial, the rats were administered a lethal dose of pentobarbital (150 mg/kg, i.p.) and transcardially perfused with 4% formaldehyde. The brains were removed and sectioned at a thickness of 50 µM, and the location of the electrode tip and cannula system was confirmed by the rapid procedure method [49]. Rats were excluded if the electrode and cannula were outside of the nuclei.

### Statistical analysis

The data are expressed as the mean ± SEM or as a percentage of the control value. Statistical significance was determined by Student´s t-test for paired data, and *P* < 0.05 was considered significant. Origin 8 graphing and statistical software (OriginLab, Northampton, MA) was used. The effect of pallidal injection on the spiking pattern in the RTn was analyzed by the burst index (BI), which was calculated by dividing ISIs < 10 ms by ISIs < 200 ms. Likewise, power data are expressed as the mean between 5 and 50 Hz, and the data from all experiments were normalized. Coherence analysis was used to assess the relationship between MCx activity and RTn activity in the same frequency ranges with a confidence level of 95% [32, 51, 77].

## Results

**Fig. 1:**
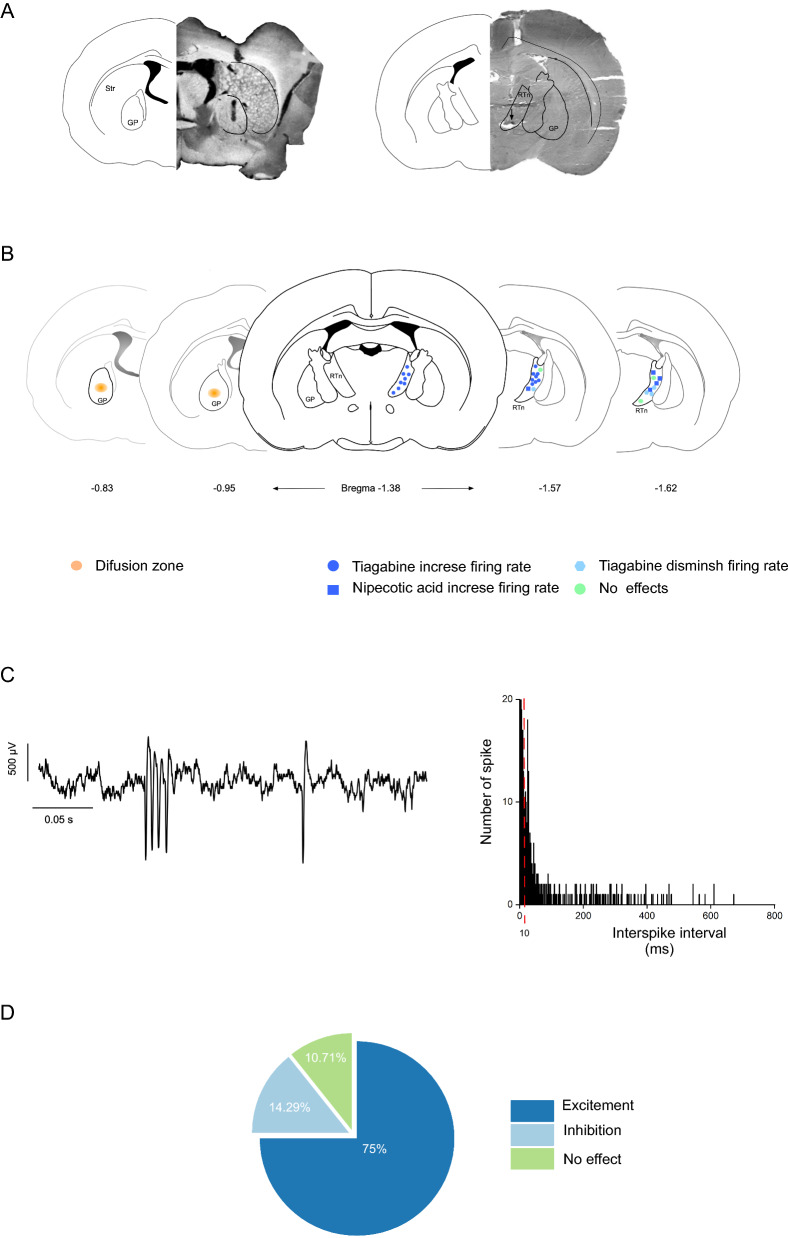
Recording locations and firing pattern characteristics within the reticular thalamic nucleus (RTn). **A** Histological verification of infusion region (right) and recording tip (left) in the coronal plane. **B** Schematic representation in the coronal plane of the recording location and response of RTn neurons. **C** Raw trace illustrating of characteristic firing pattern of the RTn neuron is shown (right). Examples of ISI histograms of one neuron recording (left). **D** Graph illustrating in percentage the response of RTn neurons

To elucidate the electrophysiological effects of pallidal GABA levels on reticular neurons and cortical activity, we pharmacologically blocked GAT-1 in the GP and recorded the spontaneous spiking activity of neurons localized to the rostral portion of the RTn in a total of 34 rats. (One neuron per rat. Four rats were excluded.) All recorded neurons displayed alternation between tonic and burst firing (*n* = 34 neurons; mean off firing rate = 7.88 ± 2.44 spikes/s); in this irregular spiking pattern (Fig. 1B), the mean BI and CV were 0.36 ± 0.16 (0.07–0.65 range) and 1.07 ± 0.49, respectively.

### GAT-1 inhibition in the external GP increases the firing rate in the RTn

**Fig. 2:**
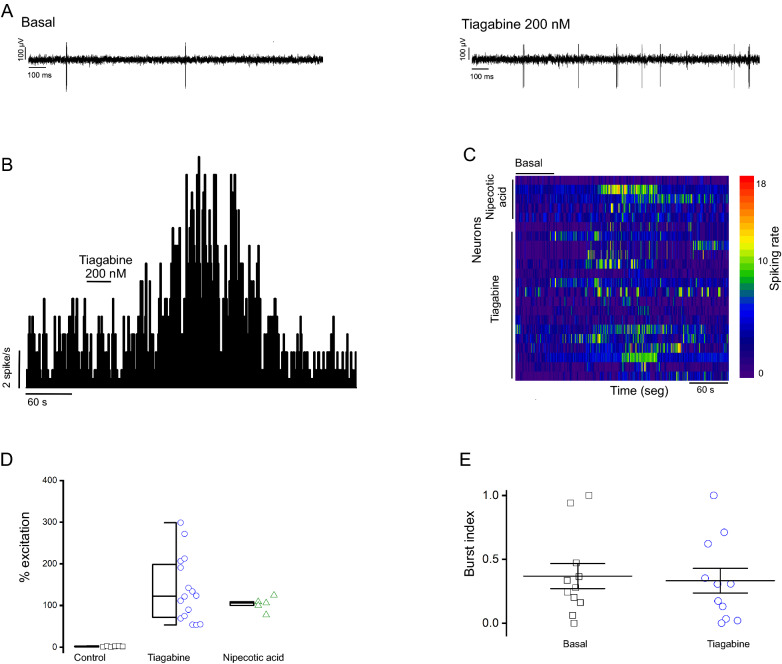
Intrapallidal tiagabine increase reticular neurons’ (RTn) spiking rate without changes in their firing pattern. **A** Raw traces illustrating firing activity in both basal (left) and tiagabine (right) conditions; voltage and time scales apply to both traces. **B** Frequency histogram of the effect of intrapallidal tiagabine (200 nM) on RTn same cell as in **A**. **C** Heatmap of the spiking rate of individual reticular neurons before and posterior to pallidal infusion of both GAT-1 antagonists. **D** Statistics the effect of tiagabine applied in the ipsilateral globus pallidus on RTn neurons spiking rate (*P < 0.05 vs. control, paired Student´s *t*-test; *n* = 16 neurons). E. Statistics of burst index analysis, each symbol represents a neuron

Tiagabine microinjection into the GP increased the firing rate of most recorded RTn neurons. Ipsilateral infusion of 200 nM tiagabine increased the spontaneous spiking rate by 138.02 ± 19.37% in 16 neurons in the RTn. The increase in firing rate started 28.13 s (at mean) after the infusion. The maximum peak was at 52.4 s, and its duration was a mean of 129.8 s. At the same dose, tiagabine diminished the firing rate of four neurons by 58.65 ± 6.45% and did not have any effect on the firing rate of three neurons. The BI and CV of RTn neurons for which the firing rate was increased by 200 nM tiagabine were 0.29 ± 0.07 and 0.87 ± 0.53, respectively. However, these values were not significantly different from the basal BI and CV of RTn cells (0.33 ± 0.06 and 0.84, respectively; *p* < 0.71; paired Student´s *t*-test; *n* = 16 neurons; Fig. 2).

Recordings were obtained from six neurons after intrapallidal administration of 100 nl of NaCl (0.9% w/v) as controls, and no changes in spiking rate and electrophysiological features were observed (spiking rate: 7.95 ± 1.36 spike/s (basal) vs. 8.1 ± 1.26 spikes/s (NaCl); BI: 0.41 ± 0.19 (basal) vs. 0.37 ± 0.28 (NaCl); CV: 0.33 ± 0.23 (basal) vs. 0.35 ± 0.5 (NaCl); *p* < 0.64; paired Student´s t test; *n* = 6 neurons).

In another set of experiments, we analyzed the effect of intrapallidal administration of nipecotic acid on the spiking rate in the RTn. Administration of 300 nM nipecotic acid into the GP increased the firing rate of five RTn neurons by 103.26 ± 7.56%; none of the neurons showed a change in the BI or CV after administration of nipecotic acid at this dose (BI: 0.41 ± 0.16 (basal) vs. 0.44 ± 0.16 (nipecotic acid); CV: 0.38 ± 0.15 (basal) vs. 0.33 ± 0.12 (nipecotic acid)).

### Inhibition of GAT-1 in pallidal neurons decreases the power spectra in the beta frequency band of ECoGs

**Fig. 3:**
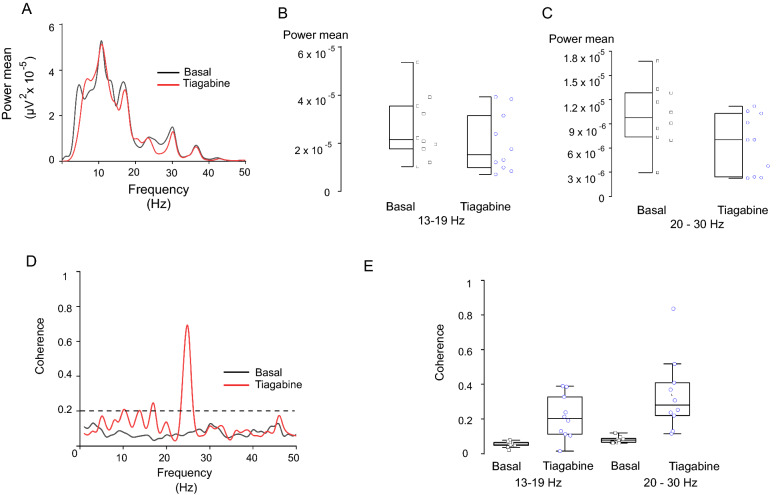
Intrapallidal inhibition of GAT-1 decreases the power spectra in the beta frequency band. **A** Power spectrum showing the effect of intrapallidal inhibition of GAT-1 in the 13 to 30 Hz frequency. **B** Statistics the effect of tiagabine applied in the ipsilateral globus pallidus on low (13–19 Hz; *p* < 0.012; paired Student´s *t*-test; *n* = 10 neurons) and high beta band (20–30 Hz; *p* < 0.027; paired Student´s *t*-test; *n* = 10 neurons). **C** Plot showing significantly coherent activity between motor cortex and RTn at frequencies associated with the beta activity. Dashed lines are 95% confidence intervals. **D** Graph showing coherence values of tiagabine at frequencies associated with the low and high beta activity

Elevation of endogenous GABA levels in pallidal neurons via inhibition of GAT-1 with 200 nM tiagabine significantly diminished the mean power spectra in the beta frequency band by 59.89 ± 11.39% relative to basal values (mean power: 1.69 µV^2^ × 10^–5^ ± 2.88 µV^2^ × 10^–6^ vs. 1.27 µV^2^ × 10^–5^ ± 2.44 µV^2^ × 10^–6^ power mean of tiagabine; paired Student´s *t*-test *p* < 0.0025; *n* = 12 neurons Fig. 3). Direct analysis of both beta frequencies (low and high) showed a significant decrease of the same magnitude. In the low beta band (13–19 Hz), the mean power value was 2.63 µV^2^ × 10^–5^ ± 4.30 µV^2^ × 10^–6^; after intrapallidal administration of tiagabine, the mean power value was 2.00 µV^2^ × 10^–5^ ± 3.91 µV^2^ × 10^–6^ (*p* < 0.012; paired Student´s *t*-test; *n* = 10 neurons Fig. 3B). The mean power in the high beta band (20–30 Hz) after local infusion of tiagabine ranged from 9.98 ± 1.23 µV^2^ × 10^–6^ to 6.60 ± 1.15 µV^2^ × 10^–6^ (*p* < 0.027; paired Student´s *t*-test; *n* = 10 neurons; Fig. 3C).

Likewise, blockade of GAT-1 by tiagabine administration into the GP significantly increased the coherence at the same frequency band between cortical and RTn activity (*p* < 0.029; paired Student´s *t*-test; *n* = 10 neurons). Coherence in the low beta band showed a significant peak, although small, compared to baseline (mean coherence: 0.05 ± 0.017 (basal) vs. 0.21 ± 0.1 (tiagabine) [peak: 0.38 ± 0.2]; *p* < 0.028; paired Student´s *t*-test; *n* = 10 neurons). A major peak (0.83 ± 0.09) in the mean coherence was observed in the high beta band after pallidal GAT-1 was blocked (mean coherence: 0.08 ± 0.04 (basal) vs. mean 0.33 ± 0.2 (tiagabine); *p* < 0.028; paired Student´s t test; n = 10 neurons; Fig. 3D, E).

**Fig. 4:**
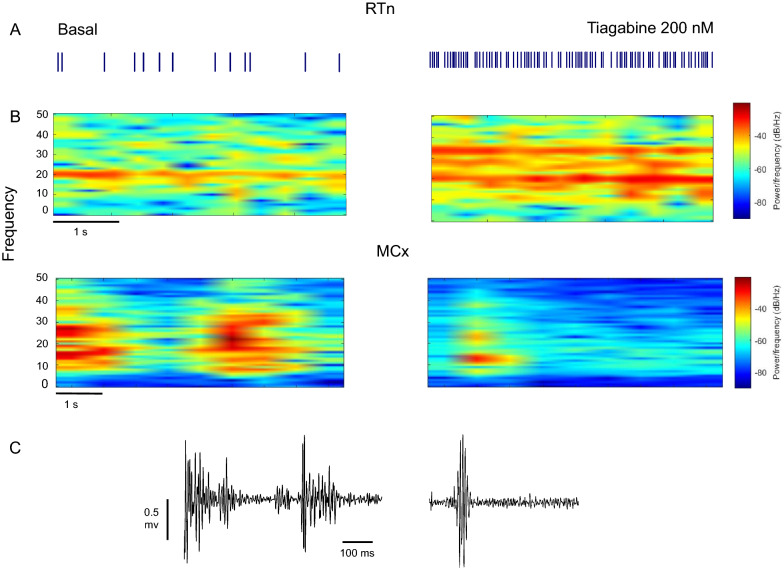
The increase in the firing rate of reticular neurons decreases cortical beta activity. The plot was derived from data of the same cell. The baseline condition is shown on the left and the tiagabine (200 nM) condition on the right. **A** Raster plots of activity of reticular cell before and during intrapallidal inhibition of GAT-1 by tiagabine. **B** Spectrograms showing the activity of reticular neurons (top) and motor cortex (bottom) at the beta frequency band. **C** Raw traces illustrating cortical activity in both basal and tiagabine conditions. Time scales apply to both traces

The simultaneous recording of the RTn and the motor cortex activity showed that the increase in the firing frequency of reticular neurons by blockade of pallidal GAT-1 decreased cortical oscillations in the beta frequency range (Fig. 4). Under basal conditions, the spontaneous firing of RTn was located at the frequency of 20 Hz (range 17 -24 Hz, Fig. 4A, B), and the cortical activity was located at 20.76 Hz (range 19.3—23.12 Hz, Fig. 3B, C). After pallidal GAT-1 inhibition, the increase in the firing frequency of RTn was located at the frequency of 23.67 (range 19.66–29.24 Hz), and the cortical oscillations were located at the frequency of 14.28 Hz (range 10.13–18.62, Fig. 4 left).

The power spectra of the MCx exhibited similar changes after microinjection of nipecotic acid into the GP. The mean power in five EcoGs obtained under basal conditions was 1.02 µV^2^ × 10^–5^ ± 2.09 µV2 × 10^–6^; after intrapallidal administration of 300 nM nipecotic acid, the mean decreased significantly to 4.35 µV^2^ × 10^–6^ ± 8.88 µV^2^ × 10^–7^ (p < 0.00007; paired Student´s *t*-test; *n* = 5 neurons). The coherence between RTn and the cortex after intrapallidal administration of 300 nM nipecotic acid was also significant in the beta band (mean coherence: 0.08 ± 0.05 (basal) vs. 0.13 ± 0.11 (nipecotic acid); *p* < 0.0009; paired Student´s *t*-test; *n* = 5 neurons). However, the coherence only showed a major peak in the high beta band (0.68 ± 0.19).

EcoGs of control neurons in the MCx did not show changes in the mean power spectra following pallidal infusion of 100 nl of NaCl (2.03 µV^2^ × 10^–5^ ± 3.62 µV^2^ × 10^–6^ (basal) vs. 2.06 µV^2^ × 10^–5^ ± 3.47 µV^2^ × 10^–6^ (NaCl); *p* < 0.9; paired Student´s t test; n = 6 neurons). In addition, signals recorded from the RTn and MCx did not show a relationship in any frequency band (coherence: 0.10 ± 0.06 (basal) vs. 0.09 ± 0.06 (NaCl); *p* < 0.25; paired Student´s *t* test; *n* = 6 neurons).

## Discussion

Through the present results, we show that pharmacological elevation of endogenous GABA levels in the GP alters the spontaneous firing rate in the RTn; this modulation results in desynchronization of the beta frequency band in the MCx.

Modulation of BG activity by the GP is mediated by two factors, i.e., GABAergic projections from the GP to different nuclei and firing frequency [44]. At this sense, it has been reported that GAT-1 is expressed in pallidal neurons and modulates their firing frequency [8, 22, 37]. However, there are few reports of the effect of GAT-1 on pallidal projections, thus, since GAT-1 is present in pallidal neurons and existence the pallido-reticular network, we decided to pharmacologically block GAT 1 into the GP and analyze the effect on RTn activity. As a result, intrapallidal application of a selective GAT-1 antagonist increased the firing frequency of reticular neurons. Despite the fact that we did not observe a direct effect of GAT-1 blockade on pallidal neurons under our experimental conditions, we associate the effect of GAT-1 blockade with the inhibition of pallidal neurons since it has been consistently shown that GAT-1 blockade inhibits the firing of GP neurons [8, 22, 37].

GATs control the extracellular levels of GABA and modulate transmission in the network [74]. In the GP, GAT-1 is located in terminal axons in striatopallidal synapses, and pharmacological blockade of GAT-1 increases the duration of synaptic currents mediated by GABA A receptors and induces persistent tonic inhibition [37] while reducing firing frequency [22]. It has been suggested that at high GABA concentrations, tonic currents may be mediated by extrasynaptic GABA A receptors [76]. Under these conditions, environmental GABA levels in the GP are high, and systemic blockade of GAT-1 increases the GABA level even more [21], thus stimulating extrasynaptic GABA A receptors in dendrites [44]. This explains the effect we observed; tonic activation of these receptors inhibits pallidal neurons in striatopallidal synapses, which in turn reduces the inhibition of the RTn, increasing the firing frequency in this brain region.

Another potential mechanism underlying the effect of the GP on the RTn is the activation of presynaptic receptors. During phasic synaptic transmission, the elevation of GABA levels favors the spillover of GABA to extrasynaptic sites, resulting in activation of presynaptic GABA B receptors, decreased GABA release in striatopallidal synapses [8], and increased inhibition mediated by these receptors both spatially and temporally [36]. At the same time, presynaptic GABA B receptors in subthalamic terminals are activated, reducing the release of glutamate onto pallidal neurons and thus inhibiting them. This finding demonstrates that presynaptic inhibition of glutamate release is secondary to GAT-1 antagonism in the GP [38], hippocampus, and cerebellar glomerulus [54, 58]. In our study, we showed that a group of reticular neurons exhibited decreased spontaneous firing in response to an increase in pallidal GABA levels. This observation may have originated from the effect of increased tonic inhibition on the intrinsic pallidal circuit and thus reducing lateral inhibition by activating some pallidal neurons [72]. In turn, this neuronal activation inhibits a reticular neuron that exerts tonic inhibition through collaterals [68, 69] in a neighboring neuron.

Our results showed that similar to tiagabine microinjection, nipecotic acid administration into the GP increased the basal firing rate of reticular neurons. Two reports support that GABA levels are involved in the effect on RTn. First, both antagonists have high selectivity for the GAT-1 transporter [6, 45]. Second, is the high density of GAT-1 in the GP [22]. However, nipecotic acid showed a difference in the intensity of the response compared to tiagabine. The variation in the effect may be because nipecotic acid decreases the amount of GABA released since it functions as a substrate for the transporter. On the contrary, tiagabine is a non-competitive inhibitor and is not captured, thus increasing the amount of GABA released. It has been suggested that GAT-3 may be responsible for controlling GABA-mediated tonic inhibition [5]. In this sense, although nipecotic acid has a strong affinity for GAT-3 [6] and showed an effect on RTn activity, we cannot assume its participation due to the low expression of GAT-3 in the pallidal cells as well as the pharmacological behavior of nipecotic acid.

Under experimental conditions similar to ours, RTn cells show three types of spontaneous firing: high-frequency bursts, irregular firing with alternating single spikes, and short bursts and single spikes [66]. In this study, we only recorded irregularly firing neurons. The spontaneous activity of such neurons is increased after infusion of GABA into the GP [83]. For this reason, we decided to evaluate the effect of high pallidal GABA levels on these cells and the influence of changes in pallidal GABA levels by BI analysis under basal and experimental conditions. We demonstrate that the mean activation rate of these neurons but not their firing pattern is altered, as we did not observe a change in either the BI or CV. A similar effect was observed after systemic blockade of NMDA receptors [82]. We assume that these changes are not induced by urethane because under deep urethane anesthesia, both reticular [66] and pallidal cells [50] maintain stable electrical activity.

In recent years, it has been shown that the firing pattern of RTn cells allows them to determine the functional status [30, 69, 80]. In this context, despite the evidence for the heterogeneity of RTn neuron firing patterns [47, 48, 66], the irregular firing of these neurons, which was analyzed by us, has received little attention. However, it was shown that two types of inhibitory neurons generate an irregular pattern in the cerebellar cortex during tonic inhibition [34]. Our results are in line with this finding since the elevation of pallidal GABA levels modulated the mean firing frequency without inducing a change in the firing pattern; these observations suggest that tonic inhibition may contribute to rapidly altering synaptic integration in a cell population and modulate the pattern of neuronal output [34].

We obtained recordings from the rostral portion of the RTn, an area that includes the motor sector and establishes connections with both the MCx and ventrolateral nuclei (VL) [53, 67, 69, 84]. In this circuit, the RTn provides feedback inhibition of TC signals and feedforward inhibition of CT signals [53]; in addition, an increase in reticular activity inhibits its targets in the VL [30]. This dynamic allows us to suggest that an increase in the firing rate in the RTn resulting from high pallidal GABA levels inhibits the VL and increases the activity of CT neurons that directly innervate the RTn. Similar electrophysiological properties have been described under urethane anesthesia and in wakefulness; under these conditions, TC cells are active [66]. Furthermore, since urethane does not influence neurotransmission in subcortical areas [56], it is accepted as an appropriate anesthetic for analyzing both BG function and its interaction with the cortex [50, 55]. Therefore, we accept that the cortical activity observed in this study was not induced by urethane.

Tiagabine increases the beta frequency on electroencephalogram (EEG) under different application conditions [11, 16, 46]. However, contrary to expectations, we found that intrapallidal administration of tiagabine decreased the beta power. A potential explanation is the reticular neurons that showed an increase in firing rate without modification of their spiking pattern, may produce short-lived PIPS in TC neurons, inhibiting them [81], thus preventing bursts that are transmitted to the cortex [57, 80] and thus decreasing beta power.

Changes in beta oscillation power have been functionally linked to phases of movement. In the premovement period, beta oscillation power is spontaneously and bilaterally reduced and subsequently increased close to the premovement period [28]. Similarly, the MCx shows a decrease in beta power during ipsilateral execution movements [42]. In humans, pharmacological blockade of GAT-1 with tiagabine during motor tasks results in an increase in beta event-related desynchronization (ERD), and a reduction in beta rebound after movement [59]. Our results provide evidence for this process since we observed a decrease in ipsilateral beta power after the pharmacological elevation of pallidal GABA levels by tiagabine, suggesting that the GP participates in the modulation of beta oscillations during phases of movement through the pallido-reticular network.

Functionally, frequencies from 3 to 10 and 11 to 30 Hz are considered predominantly anti-kinetic [73]. Consequently, desynchronization in the beta band is necessary for the initiation of movement, which is favored by increasing the firing rate of RTn cells; therefore, the effect of higher levels of pallidal GABA in inhibiting the RTn and on beta oscillations may contribute to the motor behaviors. In this respect, the effects of GAT-1 blockade in behavioral models are important in the context of our study. The application of tiagabine decreases the distance traveled and percentage of movement in an open field area [79]. In transgenic models of GAT-1 deficiency, an increase in GABAergic tonic conductance and tremor have been observed [9]. Furthermore, unilateral injection of tiagabine into the GP induces ipsilateral rotation [8], in this sense, unilateral movement of a limb produces sustained beta desynchronization [13].

However, although our results suggest that the GP exerts tonic control of the RTn and alters cortical oscillations through the pallido-reticular pathway, this phenomenon needs to be confirmed in awake animal models. In addition, factors that are known to affect neuronal responses and may have contributed to our observations, such as expression changes, desensitization, and the molecular heterogeneity of GABA A receptors involved in tonic modulation, as well as the kinetics of GATs, need to be studied. However, our results are important considering the recent finding that dopamine release is strongly modulated by GATs [71].

In conclusion, we show that elevation of pallidal GABA levels modulates the spontaneous firing of RTn neurons, in turn decreasing cortical beta power, suggesting that the GP exerts tonic control of the RTn and contributes to cortical beta oscillation dynamics.

## Abbreviations

RTn: Reticular thalamic nucleus
GP: Globus pallidus
GB: Ganglios basales
GAT: GABA transporter
MCx: Motor cortex
